# Phosphates-Containing Interpenetrating Polymer Networks (IPNs) Acting as Slow Release Fertilizer Hydrogels (SRFHs) Suitable for Agricultural Applications

**DOI:** 10.3390/ma14112893

**Published:** 2021-05-28

**Authors:** Agnieszka Lipowczan, Andrzej W. Trochimczuk

**Affiliations:** Department of Engineering and Technology of Polymers, Wroclaw University of Science and Technology Wybrzeze Wyspianskiego 27, 50-370 Wroclaw, Poland; andrzej.trochimczuk@pwr.edu.pl

**Keywords:** interpenetrating polymer networks (IPNs), full IPNs, polymer gels, hydrogels, phosphorus-containing hydrogels, hydrogels in agriculture, stimuli-responsive polymers, slow release devices, controlled release devices

## Abstract

Novel, phosphorus-containing slow release fertilizer hydrogels (SRFHs) composed of interpenetrating polymer networks (IPNs) with very good swelling and mechanical properties have been obtained and characterized. It was found that introducing organophosphorus polymer based on a commercially available monomer, 2-methacryloyloxyethyl phosphate (MEP), as the IPN’s first component network results in much better swelling properties than for a terpolymer with acrylic acid (AAc), 2-methacryloyloxyethyl phosphate (MEP) and bis[2-(methacryloyloxy)ethyl] phosphate (BMEP) when the same weight ratios of monomers are employed. The procedure described in this paper enables the introduction of much larger amounts of phosphorus into polymer structures without significant loss of water regain ability, which is crucial in the application of such materials in the agricultural field.

## 1. Introduction

Hydrogels are three-dimensional, crosslinked polymers, which are capable of absorbing and retaining significant amounts of water. Such polymers are produced from monomers containing hydrophilic functional groups, e.g., -OH, -COOH, -SO_3_H, etc. They are applied in many fields, such as: medicine, pharmacy, personal hygiene products, coatings, liquid waste treatment and others. Among possible applications, agriculture remains one of the most important, since implementing hydrogels in soil improves its water-holding capacity and promotes optimal plant growth. Hydrogels are known for their ability to increase the degree of water utilization by plants and, consequently, promote more frequent and faster germination, improving biological activity as well as increasing the biomass production. Moreover, it has been proven that applying hydrogels in agriculture supports solving problems such as soil erosion or helps alleviate the consequences of droughts [1].

Alongside appropriate irrigation, the usage of fertilizers has a critical impact on agriculture [2]. In recent years, there has been an increasing interest in the possibility of using hydrogels as controlled release devices for fertilizer delivery in order to solve the problem of their inefficient use. The combination of hydrogels and fertilizer is even described as the newest trend in research on hydrogels [3]. Such devices guarantee more effective, economical and environmentally beneficial nutrient uptake, as they prevent fertilizers from being leached from the soil [4]. Slow release fertilizer hydrogels (SRFHs) can be prepared using many different polymerization techniques, such as solution [5,6,7,8,9,10,11,12,13,14,15] or inverse suspension polymerization [16,17,18,19,20,21,22,23], as well as different mechanisms—free radical polymerization [5,6,7,8,9,10,11,12,13,14,16,18,19,20,21,22,23,24,25,26], RAFT polymerization [23], photopolymerization [15,17,26], etc. In general, there are several methods of SRFH preparation [3]. The basic two for incorporating fertilizer into hydrogel matrix are (1) adding the desired compound to the reaction mixture, then performing polymerization and (2) swelling a dry gel in the particular compound solution, thus entrapping that compound within the gel matrix, followed by drying after equilibrium of swelling is reached [13]. However, both methods exhibit some disadvantages, e.g., in the case of the former, the compound to be entrapped may influence the polymerization process and the network structure [27], while the second one requires more preparation steps [3], and thus proves to be more time and energy consuming.

The main environmental problem concerning phosphate fertilizers, essentially connected with their aforementioned washing out, is eutrophication in aquatic basins, resulting in, e.g., oxygen depletion, fish mortality and bad odors, as well as aesthetic problems [4].

**Figure 1:**
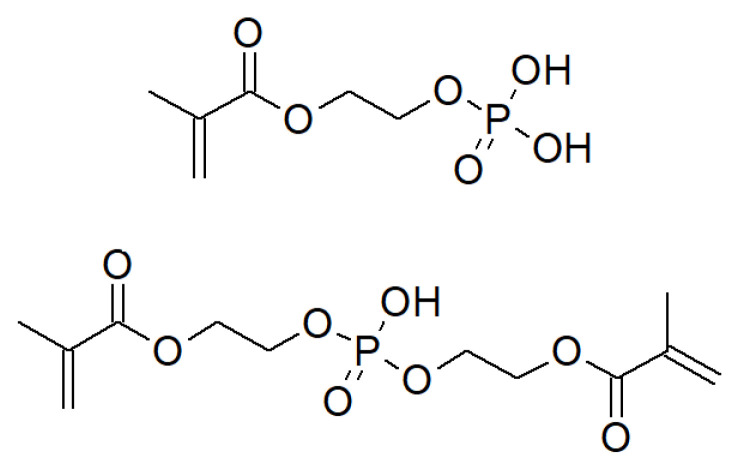
Chemical structures of monomers used: MEP (**top**) and BMEP (**bottom**).

Previously, we have reported novel hydrogels for agriculture composed of commercially available monomers: acrylic acid (AAc), phosphoric acid 2-hydroxyethyl methacrylate ester (MEP) and bis[2-(methacryloyloxy)ethyl] phosphate (BMEP) [28] (Figure 1). The presented materials exhibit very good swelling properties along with the ability to release phosphates due to organophosphorus mers hydrolysis. However, some drawbacks have been observed, e.g., relatively low phosphate content and unsatisfactory repeatability of syntheses. These limitations were not trivial to overcome—introducing more MEP and/or BMEP units was connected with a significant decrease in water regain, which, in light of agricultural applications, is highly undesirable. That led us to the change in the methodology of hydrogel preparation. In recent decades, a lot of attention has been paid to interpenetrating polymer network (IPN) hydrogels. An IPN is a combination of two or more polymers, in which at least one network is synthesized in the presence of the other, without creating covalent bonds between them. IPNs are created to confer crucial attributes of one of the networks, while maintaining the most important features of another. Sometimes, entirely new properties are obtained, which could not be observed in any of them before IPN preparation [29]. They are designed, e.g., to enhance the mechanical strength of hydrogels or their swelling/deswelling response [30]. The aim of this investigation was to prepare IPNs consisting of AAc, MEP and BMEP, with a relatively larger amount of phosphate units, but with swelling ability that still promotes their usefulness in the agricultural field. Such hydrogels may act not only as a water reservoir, but also, analogically to our previous studies, as a slow release device. This approach is advantageous in this case, as the source of phosphates is organophosphorus mers. Thus, many cost- and time-saving arguments advocate in favor of the proposed method, not to mention the ecological impact associated directly with the choice of phosphate monomers.

## 2. Materials and Methods

### 2.1. Materials

Phosphoric acid 2-hydroxyethyl methacrylate ester (MEP), bis[2-(methacryloyloxy)ethyl] phosphate (BMEP) and 2,2′-azobis(2-methylpropionamidine) dihydrochloride (AAPH) were obtained from Sigma-Aldrich (Poznan, Poland). Acrylic acid (AAc) was obtained from ACROS ORGANICS (Gliwice, Poland). Potassium hydroxide and methanol were provided by Chempur (Piekary Slaskie, Poland) and POCH S.A. (Gliwice, Poland), respectively. All chemicals were used as received.

### 2.2. Preparation of Hydrogels

Interpenetrating polymer networks consisting of poly(2-(methacryloyloxyethyl) phosphate *co*-bis[2-(methacryloyloxy)ethyl] phosphate) (further as P(MEP) in its acidic—P(MEP-H^+^) or potassium—P(MEP-K^+^) form) as a first component network and poly(acrylic acid-*co*-bis[2-(methacryloyloxy)ethyl] phosphate) (2%) (further as H_AB_2.0) as a second component network were synthesized through free radical polymerization. Firstly, P(MEP) polymerization was conducted. The process was carried out in the presence of initiator, 2,2′-azobis(2-methylpropionamidine) dihydrochloride (AAPH) 1 wt.%, in reference to the weight of commercial MEP. Polymerization was carried out in the presence of methanol, the amount of which was kept at a 1:1 ratio (*wt*) to the total weight of employed MEP. Monomer, initiator and solvent were mixed together in a flask and purged with nitrogen for 10 min. The flask was then put in a water bath equipped with a magnetic stirrer. The temperature during polymerization ranged from 50 to 70 °C, and was increased by 5 °C every 15 min. Polymerization was carried out for ~24 h. The obtained polymer was washed with a large excess of methanol and then water to remove unreacted monomer and initiator, then dried at 70 °C until a constant weight was reached. Part of the obtained polymer was then converted into potassium salt by immersing it in an excess of 0.1 M KOH for at least 24 h. After that, the polymer was washed with distilled water until a neutral pH was obtained.

The second part of IPN preparation consisted of introducing the second component network, which was characterized and described in our previous paper [28]. The quantities of each component are provided in Table 1. P(MEP) was swollen in a mixture of AAc (98 wt.%), BMEP (2 wt.%), AAPH (1 wt.% in reference to the weight of monomers) and water (1:1 weight of water:total weight of monomers ratio) for 2 h. During that time, the mixture was constantly stirred. Such a relatively short duration of swelling was dictated by the well-known hydrolytic instability of phosphate compounds. However, as was observed during a series of syntheses, 2 h was enough to obtain the P(MEP) swelling equilibrium. In the course of hydrogel characterization, the phosphorus content in the IPNs remained in satisfactory agreement with theoretical values. Subsequently, the second component network was polymerized according to the methodology described in [28], the only difference being that polymerization was carried out for 24 h, as in the case of the first component network. The same methodology was also implemented to obtain referential terpolymers (poly(AAc-*co*-MEP-*co*-BMEP) in the required weight ratio, as provided in Table 1. The presence of phosphoric acid in the commercial monomer was not taken into consideration in the calculations.

Considering the relatively low water regain of the first component network, it was expected that the enhancement of the second component network mass in the polymerization mixture would negatively impact the homogeneity of the obtained material. However, as proved by the results presented in the next sections, this should not affect the materials’ performance in the agricultural field.

### 2.3. Material Characterization

#### 2.3.1. Yield of Syntheses

The yield of the terpolymers’ syntheses was calculated using the following equation:

Y = (m_p_/m_m_)·100% (1)

where m_p_ is the mass of obtained polymer, m_m_ is the mass of employed monomers. Analogically, the yield in the case of IPNs was calculated using the formula:

Y = (m_IPN_/(m_p-FCN_ + m_m-SCN_))·100% (2)

where m_IPN_ is the mass of obtained IPN, m_p-FCN_ is the mass of polymer acting as the first component network, m_m-SCN_ is the mass of monomers comprising the second component network during the course of polymerization.

#### 2.3.2. FTIR

FTIR spectra of all polymers were collected using the Bruker Vertex 70 version Fourier transform infrared spectrophotometer (Bruker, Poznan, Poland) equipped with a diamond attenuated total reflection infrared cell in the range of 4000–400 cm^−1^ at room temperature.

#### 2.3.3. Phosphorus Content

Phosphorus content within the polymer samples was determined with the molybdenum method. The known amount of polymer weighed with an accuracy of 0.0001 g was digested in concentrated sulfuric acid (ca. 20 mL). Subsequently, when the polymer was completely decomposed, the sample was cooled down to room temperature and quantitatively transferred to a 100 mL volumetric flask. Then, 5 mL of prepared solution was placed in 50 mL volumetric flask and subjected to a reaction with perchloric acid (3.5 mL), freshly prepared amidol solution (4.5 mL) and ammonium molybdate (3.5 mL). The flask was filled with distilled water up to the calibration mark. After 20 min, UV–Vis measurements (Jasco, Cracow, Poland) were taken (*λ* = 700 nm). The amount of phosphorus was then determined based on a standard curve.

#### 2.3.4. Hydrolytic Stability

The hydrolytic stability of IPNs was examined by immersing 0.1 g of IPN sample in 150 mL of distilled water and measuring the pH with the CPC-551 Microcomputer pH and Conductivity Meter (Alchem, Wroclaw, Poland). Simultaneously, the weight of swollen sample over time was controlled with an accuracy of 0.0001 g. After changes in pH were no longer observed, the phosphorus content in solution was determined using the method described in Section 2.3.3 (without sample digestion).

The hydrolytic stability of P(MEP-H^+^) and P(MEP-K^+^) was also investigated (pH and water regain control over time).

#### 2.3.5. Water Regain

To determine the polymers’ ability to absorb water, water regain (W in [g of water/g of polymer]) tests were conducted. The polymer sample was immersed in water for at least 24 h, so the maximum swelling was obtained. After this time, the excess water was removed by filtration of hydrogels under reduced pressure for exactly 5 min using a fritted-glass funnel. Swollen hydrogel was then weighed with an accuracy of 0.0001 g and dried at 70 °C until a constant weight. After that, the weight of dry hydrogel was determined.

Water regain (W [g of water/g of polymer]) was calculated using the following equation:

W = (m_s_ − m_d_)/m_d_ (3)

where m_s_ is weight of swollen hydrogel after vacuum filtration (g) and m_d_ is the weight of dry hydrogel (g).

#### 2.3.6. Swelling Studies under Different Conditions

As in the case of previously reported materials [28], the IPNs described herein were expected to exhibit diverse properties depending on the presence of external stimuli. Since these polymers were composed of acidic units able to dissociate above their pKa (pKa_1_ and pKa_2_ of phosphoric acid are 2.15 and 7.2, respectively and pKa of acrylic acid is 4.25), it was expected that their swelling properties would change when placed in solutions of different pH. Furthermore, knowing that acidic groups within the polymer may also be sensitive to the presence of mono- and multivalent ions and their concentration, different conditions were tested. All solutions were prepared as previously described [28]. Water regain was determined using Equation (3). Based on the results presented in Table 2, only two representative samples have been chosen and deeply investigated—the one with the best swelling properties and that with the highest phosphorus content.

#### 2.3.7. Mechanical Studies

Mechanical properties of the chosen hydrogels were determined using a Shimadzu Universal Testing Instrument EZ Test kindly supplied by Shim-Pol (Warsaw, Poland). Compression tests were carried out for at least 10 samples of each hydrogel. For the compression test, swollen hydrogel particles of 6.0 ± 2.0 mm in height were selected to obtain hardness values for each hydrogel. Hardness is herein considered as a value where sample breaking was observed. Tests were conducted by employing the following parameters: analysis mode—texture, maximum force—50 N, compressing speed—25 mm/min.

## 3. Results and Discussion

### 3.1. Yield and Phosphorus Content

The yield of syntheses and phosphorus content determined as described in Section 2.3.2 and Section 2.3.3, respectively, are shown in Table 2. The yield of syntheses increased when P(MEP-K^+^) was employed as the first component network instead of P(MEP-H^+^) and this is the main reason for using it in most cases. Such differences are the result of the higher water regain of P(MEP-K^+^), which grants better monomer distribution during the swelling phase and may have a significant impact from the economical perspective, if such materials were to be produced on a large scale. The amount of incorporated phosphorus remained in good agreement with theoretical values, as P (mmol/g) values of P(MEP-H^+^) and P(MEP-K^+^) used in IPN syntheses were 3.29 ± 0.16 mmol/g and 2.36 ± 0.29 mmol/g, respectively.

### 3.2. Swelling Studies

#### 3.2.1. Water Regain

**Figure 2:**
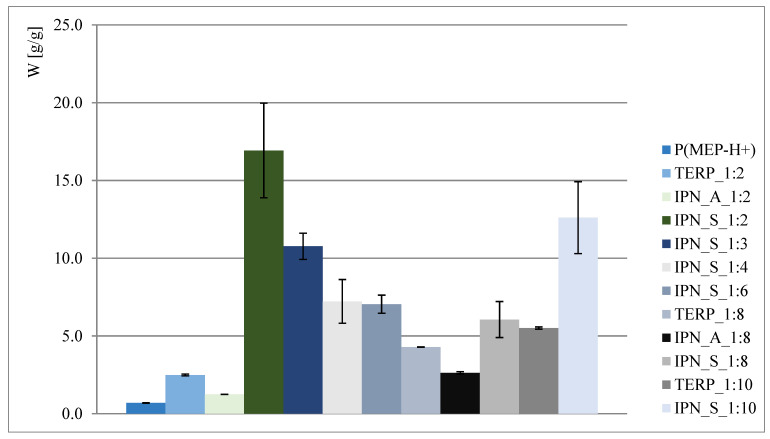
Water regain of polymers in acidic form.

**Figure 3:**
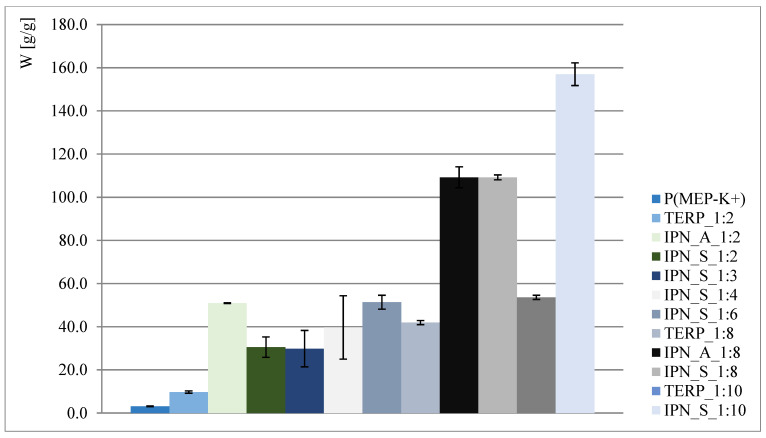
Water regain of polymers in potassium salt form.

The obtained results are presented in Figure 2 and Figure 3. As shown, interpenetrating polymer networks in the form of potassium salt exhibit much better swelling properties than their compositional analogs—terpolymers. Better swelling of samples with a higher phosphorus content (such as in the case of IPN_S_1:2, where data presented in Figure 2 might suggest that the higher the P content, the better the ability to absorb water) is probably associated with the ionic form of the first component network, so the claim that phosphate groups positively affect swelling properties is therefore untrue. This is also reaffirmed when samples where P(MEP-H^+^) acts as a first component network are taken into consideration (clearly visible when IPN_S_1:2 and IPN_A_1:2, Figure 2, are taken into consideration). However, the initial crosslinking degree, which is directly associated with BMEP content, is much higher than in most of the commercial hydrogels, yet the materials continue to exhibit very good swelling properties. This is significant, since a higher crosslinking degree influences the mechanical properties of hydrogels (further information in Section 3.5).

#### 3.2.2. Swelling under Various Conditions

**Figure 4:**
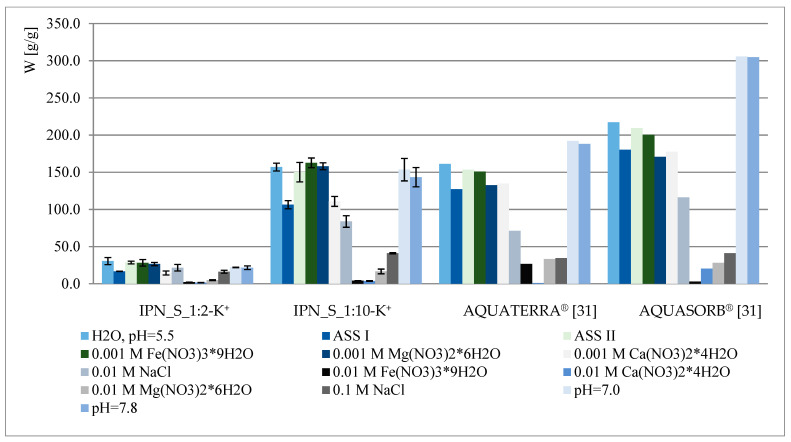
Swelling properties of chosen IPNs and commercial hydrogels under different conditions.

The water regain of selected samples under different pH and ionic strength conditions is presented in Figure 4. The obtained results were also compared with the behavior of some of the most popular agricultural hydrogels—AQUATERRA^®^ and AQUASORB^®^ (SNF Korona JV, Warsaw, Poland) [31]. Similarly to hydrogels reported previously [28], several factors affect IPN behavior, i.e., ionic strength and the type of metal present (valence, affinity to carboxyl/phosphate groups), pH and crosslinking degree/amount of phosphates within the polymer. As can be observed in Figure 4, the material presented here—IPN_S_1:10—exhibits very satisfactory properties when compared to commercial hydrogels, however, it does not possess acrylamide within its structure, which is known for its potentially carcinogenic and toxic properties [32,33,34,35]. Thus, IPN_S_1:10 may be considered a good candidate for acrylamide-based hydrogel replacement, especially when its dual role (as a water and phosphate reservoir) is taken into account. As can be concluded from the results presented in Figure 4, the water regain decrement is not as drastic here as in the case of previously reported materials (especially the ones with very low phosphate content). The increase in swelling at pH = 7.0 and 7.8, contrary to the behavior of commercial hydrogels, is not observed since all of the acidic units were converted into the potassium salt form.

### 3.3. Hydrolytic Stability

**Figure 5:**
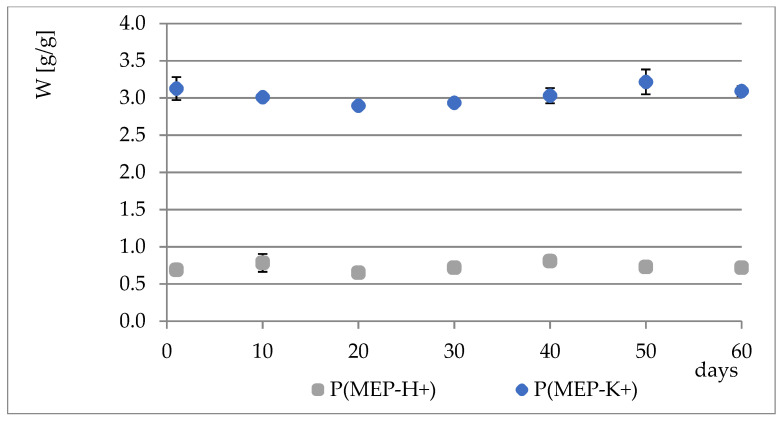
Phosphorus content changes in PMEP over a two-month period.

**Figure 6:**
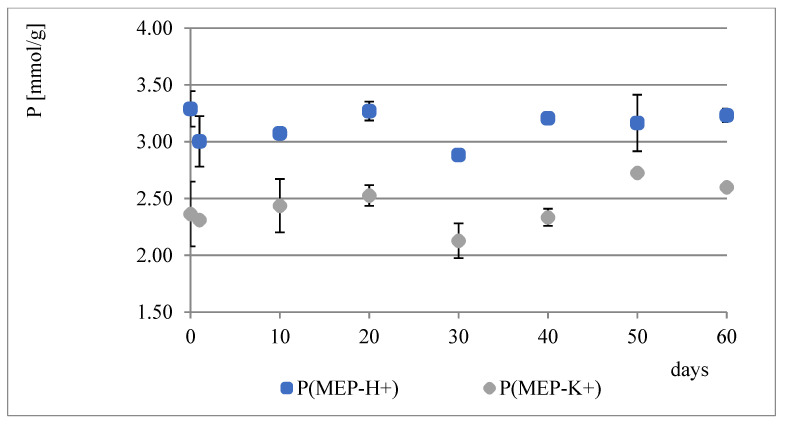
Water regain changes in PMEP over a two-month period.

**Figure 7:**
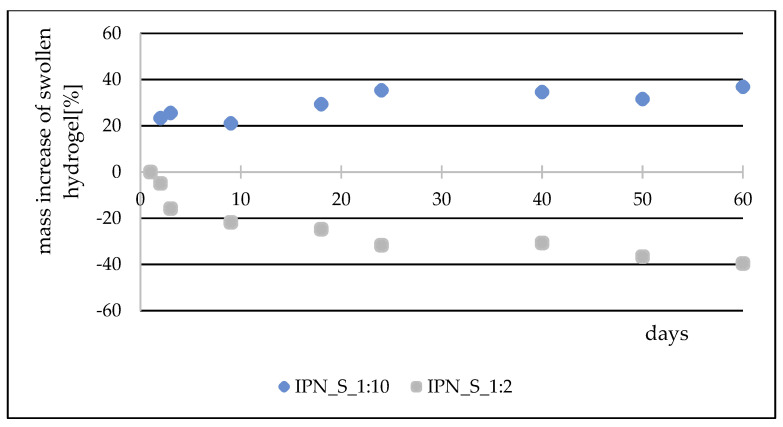
Mass changes over a two-month period for chosen IPNs.

**Figure 8:**
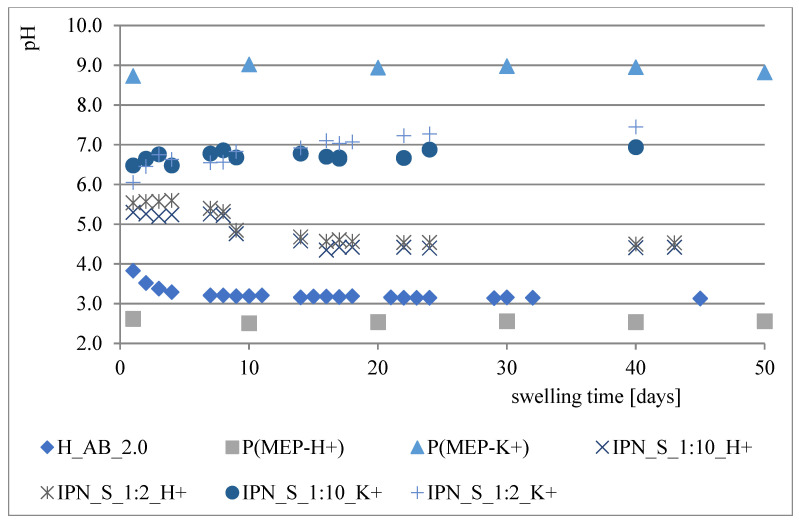
Changes in pH of the solutions above particular polymers.

During the course of our previous studies [28], it was observed that hydrogels composed of AAc, MEP and/or BMEP exhibit the ability to release phosphates, which manifests in a pH decrease and, simultaneously, an increase in swelling (as a result of a lower crosslinking degree). This phenomenon may be of great use, since the hydrogels could be applied in agriculture not only as water reservoirs, but also as an additional source of phosphorus. However, the amount of phosphorus within polymers was very small. Therefore, one of the main objectives for conducting these studies was to prepare materials with relatively higher phosphate content without losing their ability to absorb water, which seemed to be impossible to overcome in the course of free radical copolymerization of AAc, MEP and/or BMEP. From Figure 5, Figure 6, Figure 7 and Figure 8, several findings arise, e.g., the first component network, PMEP, regardless of ionic form, does not seem to hydrolyze in water. The slight change in pH above the polymer is observed in the early stages of swelling (1st day—pH = 2.62, 10th day—pH = 2.51). Additional confirmation is provided by the analysis of phosphorus content within the sample, determined using a methodology described in Section 2.3.3 (Figure 5). Such behavior does not resemble that of the second component network (H_AB_2.0, Figure 8, data from [28]). The prepared IPNs exhibit properties which may be considered intermediate ones, e.g., pH changes are observed (the increase in pH is probably the effect of the salt form of the first component network), however, the swelling properties do not improve over time or at least not as effectively as in the case of previously described SAPs (Figure 7). It has been shown previously that significant changes are easily observable only in the case of slightly crosslinked samples, which is also confirmed here. However, once pH decrease was no longer observed, the phosphorus content in solution above IPN gels was determined: 8.0 mol% of phosphorus was released from IPN_S_1:2, whereas from IPN_S_1:10, it was 90.9 mol% (based on average phosphorus content within hydrogels), which explicitly confirmed that IPNs can be applied in agriculture as a source of phosphates. Research on how they actually affect crops is now in progress.

### 3.4. FTIR

**Figure 9:**
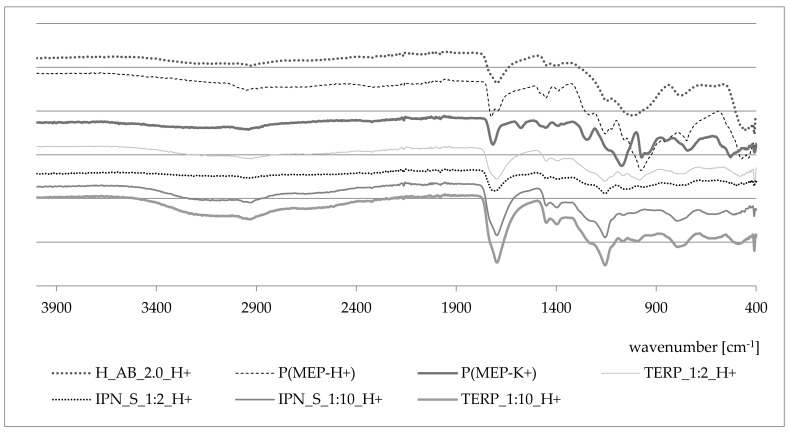
FTIR spectra of the first component network in two ionic forms, the second component network, IPNs and their corresponding terpolymers.

FTIR spectra are presented in Figure 9. The spectra of PMEP have been described in detail by L. Grøndahl et al. [36]. In Figure 9, the P(MEP-H^+^) spectrum is presented (-P-OH—974 cm^−1^, -P-OC—1057 cm^−1^, -CH_2_—1452 cm^−1^), as well as those of P(MEP-K^+^) and H_AB_2.0. The change in the ionic form of PMEP slightly affected the position of particular peaks. The -C = O peak oscillates in the ~1700 cm^−1^ region. Spectra of obtained IPNs and their compositional analogs show no significant differences compared to the expectations.

### 3.5. Mechanical Studies

Hydrogels are soft and easily deformable materials, depending on coil density, solvent content, degree of crosslinking, chemical structure, etc. For example, with a higher crosslinking degree, polymers become more brittle and harder, as the possibility of chain segments movement becomes lower. The elasticity is a characteristic property of gels with low crosslinking degree, since the deforming force is not sufficient to cause a permanent deformation. Hydrogels have worse mechanical strength than polymers in a dry state because the solvent isolates polymeric chains from each other [37]. Another important factor affecting mechanical properties is heterogeneity of the hydrogel network—when a force is applied on such a material, the stress is concentrated around the shortest chains, which can result in a failure of the sample, even when very low forces are applied [38]. Poor mechanical properties of hydrogels are a key limitation for their use in many applications, especially in the biomedical field, where they are expected to exhibit properties similar to, e.g., bone tissues. However, it is hard to obtain hydrogels with mechanical behavior comparable to that of typical engineering materials, since their most substantial fraction is water [39].

Good mechanical properties are required not only in the biomedical field, but also in agriculture, along with high permeability, biocompatibility and excellent swelling [40]. Direct mixing of hydrogels with soil remains one of the most common methods of employing SAPs in agriculture, mostly due to its simplicity [41,42,43]. Thus, mechanical properties of agricultural hydrogels are crucial, since these materials are affected by a pressure exerted by the soil layer and the plant root systems’ pressure on them [44]. As was described repeatedly in the literature [41,43,45,46,47], the water-holding capacity of superabsorbent hydrogels may decrease significantly after mixing them with soil due to the pressure of soil particles. To overcome the negative effects of mixing hydrogels with soil, a so-called geocomposite—a spatial structure consisting of nonwoven fabric, the inner armature and SAP placed inside—was proposed [47]. Nevertheless, the mechanical properties of hydrogels can be improved by a deliberate choice of monomers or by inducing physical crosslinks together with covalent bonds, which ultimately influence the topology of the network [40]. However, incorporating more hydrophobic units and/or increasing the crosslinking degree results in worse swelling properties, which is understandably a huge limitation, especially in the agricultural field, as the most important feature of SAP is no longer fulfilled.

According to the literature reports, the synthesis of IPNs is proven to be an effective method for mechanical property enhancement [38,48,49,50]. The improved mechanical properties of IPN hydrogels originate from the unique combination of two networks with opposite structures. During a deformation, the first, rather brittle, network breaks into small clusters that efficiently disperse the stress around the crack tip, thus serving as sacrificial bonds. The second, more ductile, network extends significantly, thereby withstanding large deformations [38]. In order to prove that the prepared hydrogel may successfully replace the commercial ones, compression tests were conducted for materials with similar swelling properties: AQUASORB^®^, AQUATERRA^®^ and IPN_S_1:10_K^+^. The results are presented in Table 3.

Based on the compression test, the prepared IPN_S_1:10_K^+^ does exhibit slightly better mechanical properties. The improvement in the mechanical properties may be explained by several factors:

- The presence of HEMA and phosphate-HEMA units—according to the literature [40], ethyl methacrylate fragments impart additional strength to the network, which can be explained by the presence of hydrophobic interactions and hydrogen bonds between HEMA units; these interactions are responsible for physical crosslinking within PHEMA homopolymer [51];
- lower swelling (especially when IPN_S_1:10_K^+^ and AQUASORB^®^ are compared);
- the presence of a highly crosslinked network in the structure of the IPN.

**Figure 10:**
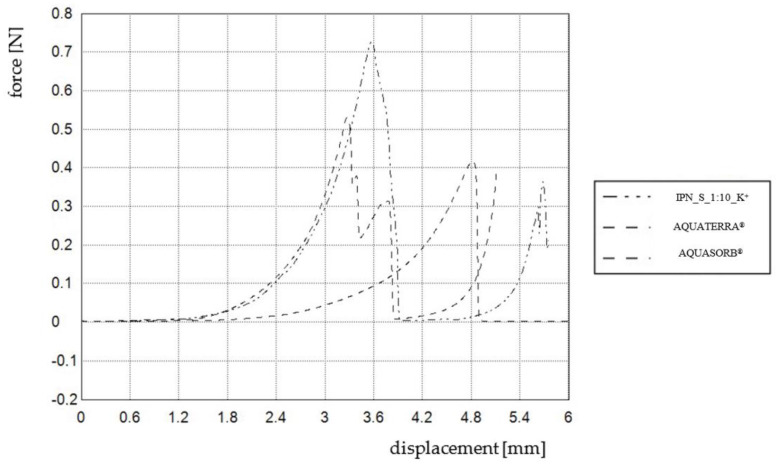
Charts presenting maximal hardness values obtained for three chosen hydrogels.

Concerning improvement, it is worth paying special attention to maximal hardness values—H_max_ of IPN_S_1:10_K^+^ is 38.5% higher than that of AQUATERRA^®^. Charts obtained during the compression tests presenting the highest hardness values are shown in Figure 10. Moreover, the compression test was also performed for IPN_S_1:8_K^+^ based on its very promising swelling performance. The results show that incorporating more phosphate units may significantly improve mechanical properties—the maximal observed hardness was 1.84 N, H_min_ = 0.19 N, while swelling properties were still good and sufficient for agricultural purposes [52].

## 4. Conclusions

Novel, phosphate-containing IPNs for agricultural applications were synthesized and characterized. The presented methodology allowed us to obtain hydrogels with much better ability to absorb water than their compositional analogs synthesized via free radical polymerization of AAc, MEP and BMEP. The IPNs exhibit properties akin to those of commercially available hydrogels, and thus could successfully replace them in the agricultural field, especially when their second function as a slow release device of phosphates is considered. In these studies, analogically to previously reported materials [28], degradation/hydrolysis processes of phosphate mers are exploited, demonstrating the materials studied here as multifunctional, smart devices, whose preparation involves only IPN synthesis. Moreover, the presented IPNs exhibit mechanical properties at least equal to the chosen commercial SAPs. This factor, combined with the presence of phosphate functional groups (known, i.a., for biocompatibility increase), may promote the compositions presented here as materials whose uses are not limited to agriculture. The fact that they are not composed of acrylamide (AAm) units is also in their favor. Depolymerization processes of PAAm commercial copolymers may lead to AAm release, which is known for its toxic/carcinogenic properties, thus applying it in the agricultural field is rather controversial. Replacing monomers such as AAm or its derivatives with less or non-toxic alternatives seems to be a proper solution.

## Figures and Tables

**Table 1:** Polymer composition. The amount of initiator and solvent was kept constant (1 wt.% in reference to monomer weight and 1:1 water:monomer weight ratio, respectively).

Polymer Code	P(MEP-H^+^) [wt.%]	P(MEP-K^+^) [wt.%]	AAc [wt.%]	MEP [wt.%]	BMEP [wt.%]
IPN_A_1:2	33.3	-	65.4	-	1.3
IPN_S_1:2	-	33.3	65.4	-	1.3
TERP_1:2	-	-	65.4	33.3	1.3
IPN_S_1:3	-	25.0	73.5	-	1.5
IPN_S_1:4	-	20.0	78.4	-	1.6
IPN_S_1:6	-	14.3	84.0	-	1.7
IPN_A_1:8	11.1	-	87.1	-	1.8
IPN_S_1:8	-	11.1	87.1	-	1.8
TERP_1:8	-	-	87.1	11.1	1.8
IPN_S_1:10	-	9.1	89.1	-	1.8
TERP_1:10	-	-	89.1	9.1	1.8

**Table 2:** The yield of synthesis and phosphorus content.

Polymer Code	Y [%]	P [mmol/g]
IPN_A_1:2	86.3	1.04 ± 0.16
IPN_S_1:2	90.8	0.82 ± 0.01
TERP_1:2	87.6	0.83 ± 0.05
IPN_S_1:3	92.5	0.65 ± 0.01
IPN_S_1:4	94.0	0.41 ± 0.00
IPN_S_1:6	93.7	0.30 ± 0.08
IPN_A_1:8	87.5	0.28 ± 0.13
IPN_S_1:8	96.4	0.32 ± 0.02
TERP_1:8	93.3	0.41 ± 0.02
IPN_S_1:10	99.7	0.10 ± 0.01
TERP_1:10	86.4	0.13 ± 0.01

**Table 3:** Hardness (H): minimum, maximum and average, obtained via compression test.

Hydrogel	H_min_ [N]	H_max_ [N]	H_av_ [N]
AQUASORB^®^	0.13	0.41	0.25 ± 0.10
AQUATERRA^®^	0.11	0.52	0.31 ± 0.13
IPN_S_1:10	0.12	0.72	0.32 ± 0.15

## Data Availability

The data presented in this study are available on request from the corresponding author.
